# Immunocytochemical Localization of Amines and GABA in the Optic Lobe of the Butterfly, *Papilio xuthus*

**DOI:** 10.1371/journal.pone.0041109

**Published:** 2012-07-23

**Authors:** Yoshitaka Hamanaka, Michiyo Kinoshita, Uwe Homberg, Kentaro Arikawa

**Affiliations:** 1 Laboratory of Neuroethology, Sokendai, The Graduate University for Advanced Studies, Shonan Village, Hayama, Kanagawa, Japan; 2 Department of Biology, Animal Physiology, University of Marburg, Marburg, Germany; Imperial College London, United Kingdom

## Abstract

Butterflies have sophisticated color vision. While the spectral organization of the compound eye has been well characterized in the Japanese yellow swallowtail butterfly, *Papilio xuthus*, neural mechanisms underlying its color vision are largely unexplored. Towards a better understanding of signal processing in the visual system of *P. xuthus*, we used immunocytochemical techniques to analyze the distribution of transmitter candidates, namely, histamine, serotonin, tyramine and γ-aminobutyric acid (GABA). Photoreceptor terminals in the lamina and medulla exhibited histamine immunoreactivity as demonstrated in other insects. The anti-histamine antiserum also labeled a few large medulla neurons. Medulla intrinsic neurons and centrifugal neurons projecting to the lamina showed serotonin immunoreactivity. Tyramine immunostaining was detected in a subset of large monopolar cells (LMCs) in the lamina, transmedullary neurons projecting to the lobula plate, and cell bodies surrounding the first optic chiasma. An anti-GABA antiserum labeled a subset of LMCs and populations of columnar and tangential neurons surrounding the medulla. Each of the four antisera also labeled a few centrifugal neurons that innervate the lobula complex from the central brain, suggesting that they have neuromodulatory roles. A distinctive feature we found in this study is the possibility that tyramine and GABA act as transmitters in LMCs of *P. xuthus*, which has not been reported in any other insects so far.

## Introduction

In many insect species, visual input dominates information about the outside world and is crucially involved in foraging, prey and mate recognition, spatial orientation, and many other behaviors. Following signal transduction in photoreceptors of the compound eyes, visual signals are processed in the optic lobes that dominate the cephalic ganglia and, in some species, contain up to 75% of all brain neurons [1]. Although the optic lobe is one of the most intensively studied areas in the insect nervous system, our understanding about its function is still quite limited.

The optic lobe is generally composed of three parts, the lamina, medulla, and lobula complex. In Diptera, the lobula complex is subdivided into the lobula, involved in color-coding mechanisms, and the lobula plate, an achromatic motion computation center [2]–[6]. Similar subdivisions are also present in Lepidoptera, Coleoptera and Trichoptera, but their physiological engagement is less well explored [7], [8].

The lamina has a retinotopic organization with modules called cartridges, each corresponding to a particular ommatidium or, in neural superposition eyes, a fixed number of neighboring ommatidia [1]. The medulla and lobula are also organized retinotopically with modules called columns, but their relationship with the ommatidia may be degraded. Medulla and lobula complex are, furthermore, organized into layers perpendicular to the columnar axes. Such orderly structure is quite amenable to the study of neural wiring. The cellular components of the optic lobe have been most thoroughly investigated in flies including *Drosophila melanogaster* [1], [9]–[15]. In addition, visual circuits in the lamina and distal medulla of *D. melanogaster* have been extensively analyzed at the electron microscopic level [16], [17].

Several lines of evidence indicate that insect neurons utilize small molecules such as acetylcholine, amines, γ-aminobutyric acid (GABA), and several types of neuropeptides as neuroactive substances. GABA is a wide-spread inhibitory neurotransmitter. Among amines, histamine is a neurotransmitter of photoreceptors [18], while serotonin (5-hydroxytryptamine, 5-HT), octopamine and tyramine are neuromodulatory substances [7], [19]–[22]. Such evidence has partly been acquired by immunocytochemistry, a powerful tool to analyze the distribution of these molecules, which can serve as an important basis for following physiological studies, and ultimately to understand how insect nervous systems function.

The Japanese yellow swallowtail butterfly, *Papilio xuthus*, has sophisticated color vision [23], including color constancy [24] and simultaneous color contrast [25]. They can discriminate wavelength differences as small as 1–2 nm in three regions of their visible light range [26] and colors of disks of about 1° in diameter, which is equivalent to the limit of their spatial resolution. This performance suggests that *P. xuthus* can resolve wavelength information with single or at least a few ommatidia [27].

To understand the basis of their color vision ability, we have analyzed the organization of the compound eye retina. The compound eye of *P. xuthus* consists of about 12,000 ommatidia. Each ommatidum contains nine photoreceptor cells (R1–R9), which are subdivided into two morphological classes; short visual fibers (R3–R8) terminating in the lamina and long visual fibers (R1, R2 and R9) terminating in the medulla [28]. All photoreceptors bear rhodopsin-containing microvilli and together form a fused rhabdom. The photoreceptors can also be divided into six classes based on spectral sensitivities: they are of the UV, violet, blue, green, red and broad-band classes. The spectral receptors are embedded in the ommatidia in three fixed combinations, making the eye a collection of three types of spectrally heterogeneous ommatidia [29].

To understand the neuronal mechanisms underlying color vision, detailed information about the anatomy of the optic lobe is indispensable. As a first step, we here performed immunostaining in the optic lobe of *P. xuthus* with antibodies against histamine, 5-HT, tyramine and GABA in order to identify cell bodies and processes of neurons containing these neurotransmitter/modulator candidates.

## Materials and Methods

### Animals

We used adult *Papilio xuthus* Linnaeus (Papilionidae, Lepidoptera) of both sexes that emerged from diapausing or non-diapausing pupae. Eggs were collected from females caught around the campus of Sokendai, Kanagawa, Japan. We reared hatched larvae on fresh citrus leaves under 10∶14-hour (diapause-inducing) or 14∶10-hour (diapause-averting) light∶dark photoperiod at 25°C. The diapausing pupae were kept at 4°C for at least 3 months before being allowed to emerge under 12∶12-hour light∶dark photoperiod at 20–25°C.

### Azur II staining

Dissected tissues were pre-fixed in 2% glutaraldehyde (GA) and 2% paraformaldehyde (PFA) in 0.1 M cacodylate buffer (CB, pH 7.3) for 30–60 minutes. After washing in 0.1 M CB, the tissues were post-fixed in 2% osmium tetroxide in 0.1 M CB for 2 hours at room temperature. The tissues were then dehydrated in a graded series of acetone, embedded in Epon via propylene oxide, and polymerized overnight at 60°C. Blocks were cut with a diamond histo-knife at 5 µm thickness and the sections were stained with Azur II according to the method described elsewhere [28].

### Immunofluorescent staining

Different fixation protocols were employed for labeling with different antibodies.

1. Rabbit polyclonal anti-histamine (Cat. No. AB5885, Chemicon-Millipore, Billerica, MA, USA): Fixed in 4% carbodiimide (E-7750, Sigma, St. Louis, MO, USA) in 0.067 M phosphate buffer (PB, pH 7.2) for 3–5 hours on ice.
2. Rabbit polyclonal anti-serotonin (5-HT) (Cat. No. 20080, ImmunoStar, Hudson, WI, USA): Fixed in 4% PFA plus 7.5% saturated picric acid in 0.067 M PB for 3 hours on ice or overnight at 4°C.
3. Rabbit polyclonal anti-tyramine (Cat. No. AB124, Chemicon-Millipore): Fixed with 2.5% GA in 0.067 M PB containing 1% sodium metabisulfite (SMB) for 4 hours on ice.
4. Rabbit anti-GABA (product No. A 2052, Sigma): Fixed in solution containing 1 part 25% GA and 3 parts saturated picric acid plus 1% acetic acid for 3–5 hours on ice.

The cornea was removed after fixation. The remaining retina and optic lobe were embedded in gelatin/albumin mixture, and then post-fixed in 7–8% PFA in PB overnight at 4°C. For tyramine labeling, 0.5% SMB was added to the PFA solution. Post-fixed tissues were sliced either frontally or horizontally with a vibrating blade microtome at 30–40 µm thickness in PB (PB with 0.5% SMB for tyramine labeling), followed by incubation in 0.25% sodium borohydride in PB for 20 minutes and subsequent wash in PB (PB with 0.5% SMB for tyramine labeling). Then non-specific binding sites were blocked with 5% normal goat serum (NGS) in 0.01 M phosphate-buffered saline (PBS, pH 7.3) containing 0.5% Triton X-100 (PBST, pH 7.3), and finally incubated for 2–3 days at 4°C in the respective primary antibodies at working dilutions listed in Table 1. The specificity of the antibodies in *P. xuthus* was confirmed by appropriate liquid-phase preadsorption tests according to the method described in Hamanaka et al [30].

**Table 1:** Antibodies used for immunocytochemistry.

Antibody	Immunogen	Working dilution	Source
Anti-histamine	histamine coupled to KLH with carbodiimide	1∶500 (fluorescence)	Cat. No. AB5885, Chemicon-Millipore
Anti-serotonin (5-HT)	5-HT coupled to BSA with PFA	1∶1,000 (fluorescence); 1∶200,000 (PAP)	Cat. No. 20080, ImmunoStar
Anti-tyramine	p-tyramine-glutaraldehyde-N-alpha-acetyl-L-lysine-N-methylamide	1∶1,000 (fluorescence); 1∶5,000 (PAP)	Cat. No. AB124, Chemicon-Millipore
Anti-GABA	GABA coupled to BSA	1∶1,000 (fluorescence)	Cat. No. A 2052, Sigma
Anti-synapsin	synapsin protein coupled to GST	1∶50 to 1∶80 (fluorescence)	3C11, DSHB

Abbreviations: BSA, bovine serum albumin; DSHB, Developmental Studies Hybridoma Bank; GABA, γ-aminobutyric acid; GST, glutathione-S-transferase; KLH, keyhole limpet hemocyanin; PAP, peroxidase-anti-peroxidase technique; PFA, paraformaldehyde.

To visualize neuropil structures, sections were simultaneously incubated with a mouse monoclonal antibody against synapsin, a presynaptic marker (SYNORF1 or antibody 3C11; Developmental Studies Hybridoma Bank (DSHB), Iowa City, IA). After washing in PBST, the sections were incubated in corresponding secondary antibodies (anti-rabbit IgG conjugated to Cy3 (1∶200; Jackson ImmunoResearch, West Grove, PA), or goat anti-mouse or goat anti-rabbit IgG conjugated to Alexa Fluor 488 (1∶200; Molecular Probes, Eugene, OR)) in PBST containing 5% NGS overnight at 4°C, followed by wash in PBST and PBS. In tyramine and GABA immunolabeling, tissues were further incubated with the nuclear marker DAPI (D9542, Sigma; 10 µg/ml in PBS) after incubation in secondary antibody. The sections were mounted in Vectashield (H-1000; Vector, Burlingame, CA).

### Peroxidase-anti-peroxidase method

For 5-HT and tyramine immunolabeling, we also applied the indirect peroxidase-anti-peroxidase (PAP) technique [31]. Procedures prior to the secondary antibody incubation were the same as described above, except for the concentration of primary antibodies. After primary antibody incubation, the sections were incubated in goat anti-rabbit IgG (1∶40, R-5506, Sigma) in PBST containing 1% NGS, and further in rabbit PAP-complex (1∶300, Dako, Denmark) in PBST. After washing in PBST, sections were developed in 0.032% 3,3′-diaminobenzidine (DAB) in 0.1 M Tris-HCl buffer (pH 7.3) containing 0.0145% H_2_O_2_ and 0.3% nickel ammonium sulfate. Sections were mounted on gelatin-coated glass slides, dehydrated in grading ethanol series, cleared in xylene, and mounted in Permount beneath cover glasses.

### Image acquisition and processing

The slices labeled with fluorescing dyes were imaged using a confocal laser scanning microscope (Fluoview FV500; Olympus, Tokyo, Japan) equipped with HC PL APO 10×/0.4 IMM CS (Leica, Germany), UPlanSApo 20×/0.85, UPlanApo 40×/1.0, and UPlanApo 100×/1.35 (Olympus) oil immersion objectives. DAPI excited with an UV-Ar laser at 351 nm was viewed through a 380–470 nm band-pass filter, Alexa 488 excited with an Ar laser at 488 nm through a 505–525 nm band-pass filter, and Cy3 excited with a HeNe green laser at 543 nm through a 560 nm long-pass filter. Confocal optical sections were acquired at an interval of 0.5 to 1.5 µm and a size of 2,048×2,048 pixels. All confocal images are stacks of 10 to 35 optical slices if not stated otherwise. DAB-labeled slices were digitally imaged with a CCD camera (DP72, Olympus) mounted on a light microscope (BX60, Olympus). Five to eight images focusing at different planes were manually taken from single slices at an interval of 3–5 µm, and then superimposed into a single image using Zerene Stacker software (Zerene Systems LLC, WA, USA). The size, contrast, and brightness of the images were adjusted using Photoshop CS4 (Adobe Systems, Tokyo, Japan) and Corel Draw X4 (Corel, Ottawa, ON, Canada).

## Results

### Neuroanatomy of the optic lobe

**Figure 1:**
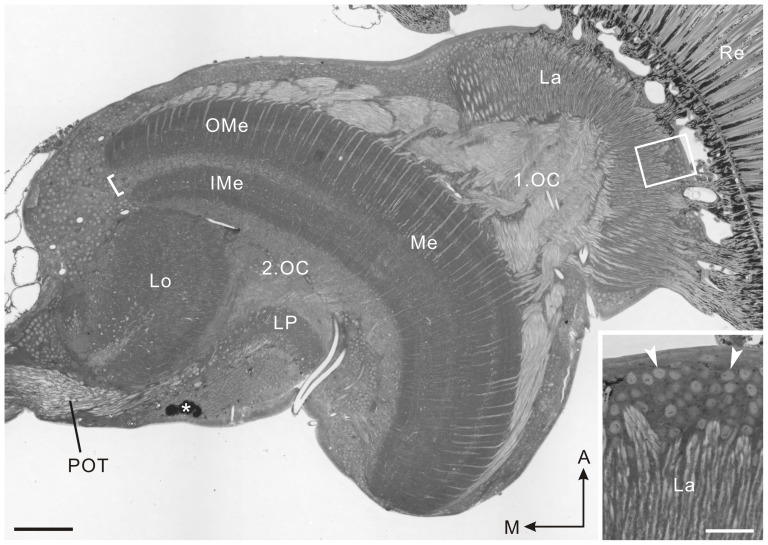
Horizontal section through the optic lobe of *Papilio xuthus*, stained with Azur II. The optic lobe comprises, from distal to proximal, the lamina (La), medulla (Me), lobula plate (LP), and lobula (Lo). The first optic chiasma (1.OC) and second optic chiasma (2.OC) connect the lamina and medulla, and the medulla and lobula complex, respectively. The medulla consists of the outer medulla (OMe) and inner medulla (IMe) separated by the serpentine layer or layer 5 (bracket). Inset shows enlarged image of a rectangle in the main panel. Cell bodies of large monopolar cells (LMCs) are densely packed in the lamina cell body layer (arrowheads). Asterisk indicates a cluster of remnant larval stemmata. A, anterior; M, medial; POT, posterior optic tract; Re, compound eye retina. Scale bar = 100 µm; 20 µm in inset.

**Figure 2:**
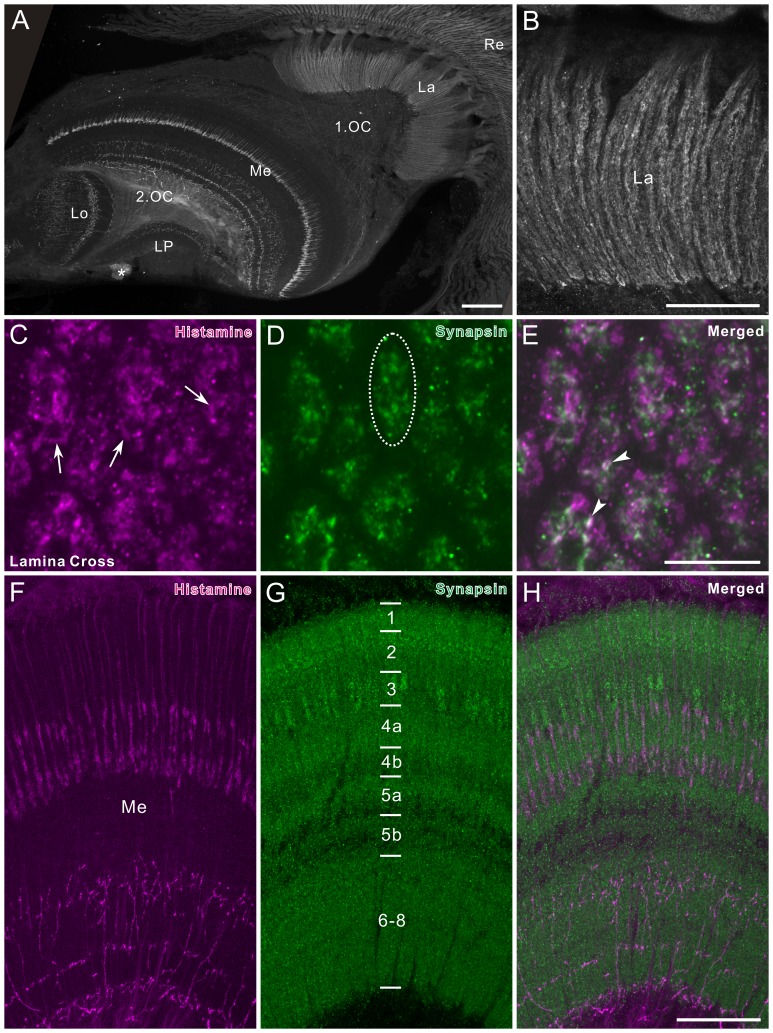
Histamine immunofluorescence in the optic lobe of *Papilio xuthus*. **A**: Horizontal section. Histamine immunoreactivity was detected in the lamina (La), medulla (Me), lobula (Lo), and lobula plate (LP). The first optic chiasma (1.OC) was weakly labeled. Asterisk indicates a cluster of remnant larval stemmata, which exhibits strong histamine immunoreactivity. **B**: Enlarged image of a part of the lamina synaptic layer in A. Short visual fiber endings express histamine immunoreactivity. **C–E**: Lamina cross section, double-labeled with anti-histamine (magenta in C,E) and anti-synapsin (green in D,E). Images are single optical sections of 0.5 µm thickness. Dotted oval in D encloses a single cartridge profile. Histamine immunostaining is distributed widely within cartridges, and in some places it is concentrated in discontinuous ring-like structures (arrows in C). Arrowheads in E point at colocalizations of histamine- and synapsin immunoreactivities. **F–H**: Horizontal section through the medulla, double-labeled with anti-histamine (magenta in F,H) and anti-synapsin (green in G,H). The numbers in G indicate medulla layers visualized by anti-synapsin labeling. Anti-histamine strongly labeled axon projections that invade layers 1–3 and terminate in layer 4 of the medulla. The terminal specializations correspond to those of long visual fibers (R1, 2 and 9). Proximal layers 6–8 are widely and sparsely supplied with numerous fine immunoreactive fibers. 2.OC, second optic chiasma. Scale bar = 100 µm in A; 50 µm in B; 10 µm in E (applies to C,D); 50 µm in H (applies to F,G).

**Figure 3:**
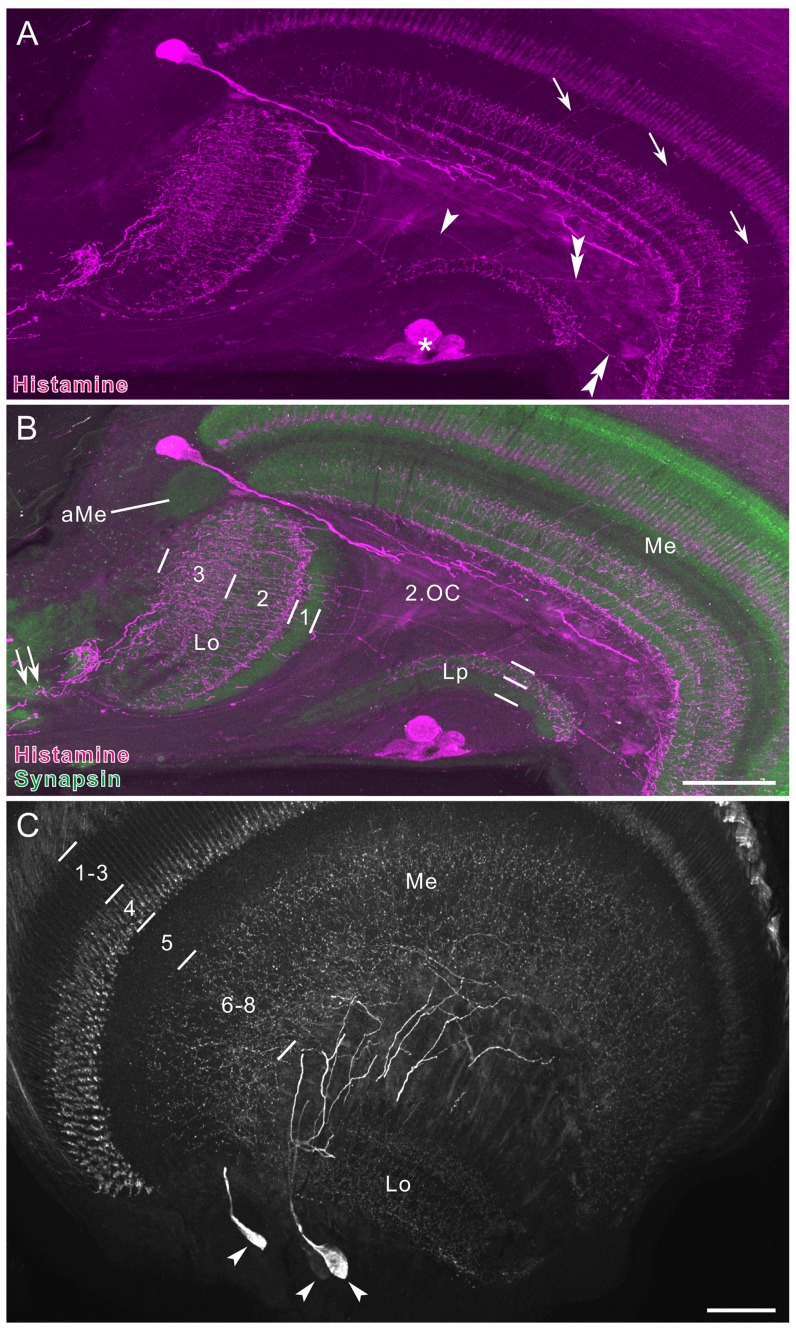
Histamine immunofluorescence in the medulla and lobula complex. **A,B**: Horizontal section, double-labeled with anti-histamine (magenta in A,B) and anti-synapsin (green in B) antibodies. A large histamine-positive neuron with cell body near the anterior rim of the medulla (Me) extends its axon into the second optic chiasma (2.OC). Proximal layers 2 and 3 of the lobula (Lo), but not layer 1 are densely innervated by processes from the central brain (double arrow in B). A few processes in the lobula pass through layer 1 and invade the outer layer of the lobula plate (LP, arrowhead in A). Double arrowheads indicate thin fibers, connecting the lobula plate to the proximal surface of the medulla. The accessory medulla (aMe) is free of labeling. In medulla layer 5, fine fibers parallel to columns are labeled (arrows in A). **C**: Frontal section through the medulla, showing three immunoreactive cell bodies at the anterior rim of the medulla (arrowheads; dorsal to the left and distal to the top). Their processes project to the proximal surface of the medulla (Me) and ramify into numerous fine fibers that innervate layers 6–8 of the medulla. Asterisk in A indicates stemmata. Lo, lobula. Scale bar = 100 µm in B (applies to A),C.

The optic lobe of *P. xuthus* comprises, from distal to proximal, the lamina, medulla, lobula plate, and lobula (Fig. 1). These visual neuropils are retinotopically connected by two optic chiasmata. The first optic chiasma links the lamina and medulla, and the second optic chiasma links the medulla and lobula complex. The lamina is composed of an array of modules called cartridges. Each cartridge is innervated by nine photoreceptors (R1–R9) originating from an ommatidium and contains processes from a few large monopolar cells (LMCs) and some unidentified neurons [28]. The medulla, lobula and lobula plate are stratified. The medulla of *P. xuthus* is divided into eight layers based on different patterns of synapsin labeling (Figs. 2G): both layers 4 and 5 are further divided into two sublayers (Fig. 2G). The lobula and lobula plate have three and two layers, respectively (Fig. 3B). The medulla, lobula and lobula plate are connected to the central brain through several tracts, including the posterior optic tract (Figs. 1). The anatomical terminology is based on that for the sphinx moth, *Manduca sexta* [32].

Here we describe the distribution of three biogenic amines, histamine, serotonin (5-HT) and tyramine, and an amino acid neurotransmitter γ-aminobutyric acid (GABA) through immunocytochemistry. Neurons and their ramifications immunoreactive to antibodies against these molecules were widely distributed in the optic lobe.

### Histamine

The anti-histamine labeled a small number of cell bodies in the optic lobe and numerous neuronal processes in the lamina, medulla, lobula, and lobula plate (Figs. 2A, and 3). The lamina synaptic layer was strongly labeled. The retina, which contains the photoreceptor cell bodies, was labeled as strongly as the lamina synaptic layer (Fig. 2A). Large histamine-positive axon terminals in the lamina (Fig. 2B) are most likely those of short visual fibers (R3–R8). Cross sections of the lamina revealed that histamine immunostaining is scattered in cartridges (Fig. 2C–E). In some places, we found discontinuous ring-like staining patterns (arrows in Fig. 2C), which probably show histamine-containing vesicles located near the photoreceptor axon membrane. Double labeling of cross sections of the cartridges revealed partial colocalization of histamine and synapsin signals (arrowheads in Fig. 2E), suggesting the assembly of synaptic vesicles containing histamine at the photoreceptor output synapses.

The histamine antiserum weakly labeled numerous fibers in the first optic chiasma (Fig. 2A). Strongly-labeled fibers in the distal portion of the medulla bear no side branches in layers 1–3, and terminate in layer 4 where they swell to give rise to blebby terminals (Fig. 2F–H). These morphological properties correspond to those of long visual fibers (R1, 2 and 9) [28]. The serpentine layer 5 is almost free of histamine immunostaining, except for some fine processes running in parallel with the columns (arrows in Fig. 3A). Layers 6–8 are sparsely innervated by histamine-immunoreactive processes, which appear to originate from a few large neurons with cell bodies in the anterior rim of the medulla (arrowheads in Fig. 3C). The accessory medulla, a small neuropil at the anterior base of the medulla, is free of histamine immunoreactivity (aMe, Fig. 3A,B).

In the lobula, the proximal layers 2 and 3 are innervated by putative centrifugal fibers derived from the central brain (double arrow in Fig. 3B). A few histamine-positive fibers extend through the distal surface of the lobula toward the lobula plate and innervate its outer layer (arrowhead in Fig. 3A). The inner layer of the lobula plate is free of anti-histamine labeling (Fig. 3B). A small number of fine processes link the medulla and lobula plate (double arrowheads in Fig. 3A). The photoreceptors of remnant larval stemmata, located posteriorly to the lobula plate, display strong histamine immunoreactivity (asterisk in Fig. 3A).

### Serotonin (5-HT)

**Figure 4:**
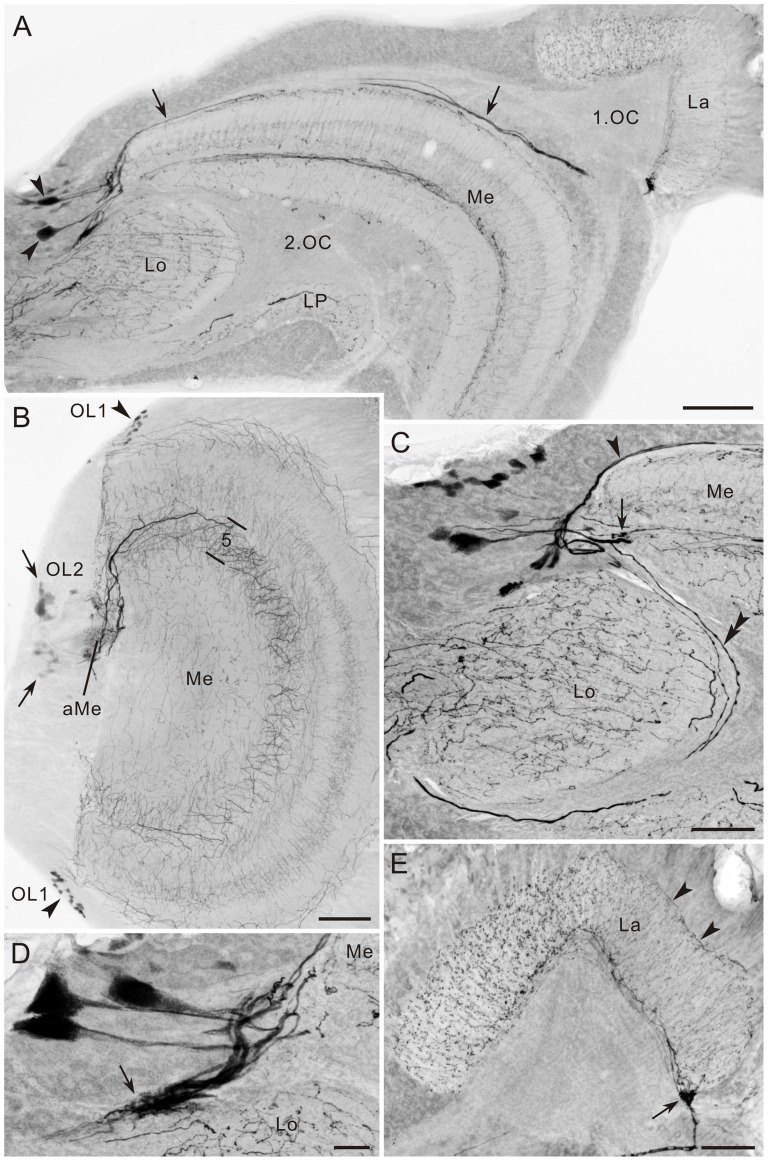
5-HT immunoreactivity in the optic lobe of *Papilio xuthus*. **A**: Horizontal section. Large immunoreactive cell bodies at the anterior rim of the medulla (Me; arrowheads) send centrifugal axons along the distal surface of the medulla (arrows) to the lamina (La). **B**: Frontal section through the medulla, showing 5-HT-positive cells located at the dorsal and ventral anterior margins of the medulla (OL1, arrowheads), and near the accessory medulla (aMe; OL2, arrows). The OL2 cells project thick fibers into medulla layer 5. The accessory medulla is innervated by 5-HT-positive fibers. **C**: OL2 cells at the anterior rim of the medulla (horizontal view, anterior to the top and medial to the left). Three distinct trajectories of processes are visible (arrowhead, arrow, and double arrowhead). **D**: Large OL2 cells, projecting fibers through the anterior edge of the lobula (arrow, horizontal view) toward the central brain. **E**: Horizontal section through the lamina (La, anterior to the left, distal to the top). Centrifugal axons from medulla OL2 neurons enter the lamina at its posterior inner edge (arrow). Numerous immunoreactive processes with blebs extend throughout the lamina neuropil, but the anterior half is more densely innervated than the posterior half. Arrowheads indicate fibers running along the distal surface of the lamina. 1.OC, first optic chiasma; 2.OC, second optic chiasma. Scale bar = 100 µm in A,B; 50 µm in C,E; 20 µm in D.

The optic lobe is widely innervated by 5-HT-positive processes (Fig. 4A). The anti-5-HT antiserum labeled three sets of cell bodies near the medulla (Fig. 4B). Two clusters of small cell bodies, OL1, are located at the anterior dorsal and anterior ventral edges of the medulla, and the third group of larger cell bodies, OL2, is located at the anterior median edge of the medulla (Fig. 4B). A similar distribution of 5-HT-positive cell bodies has been reported for the sphinx moth *M. sexta* [33]. The accessory medulla is also invaded by 5-HT-positive fibers (Fig. 4B).

**Figure 5:**
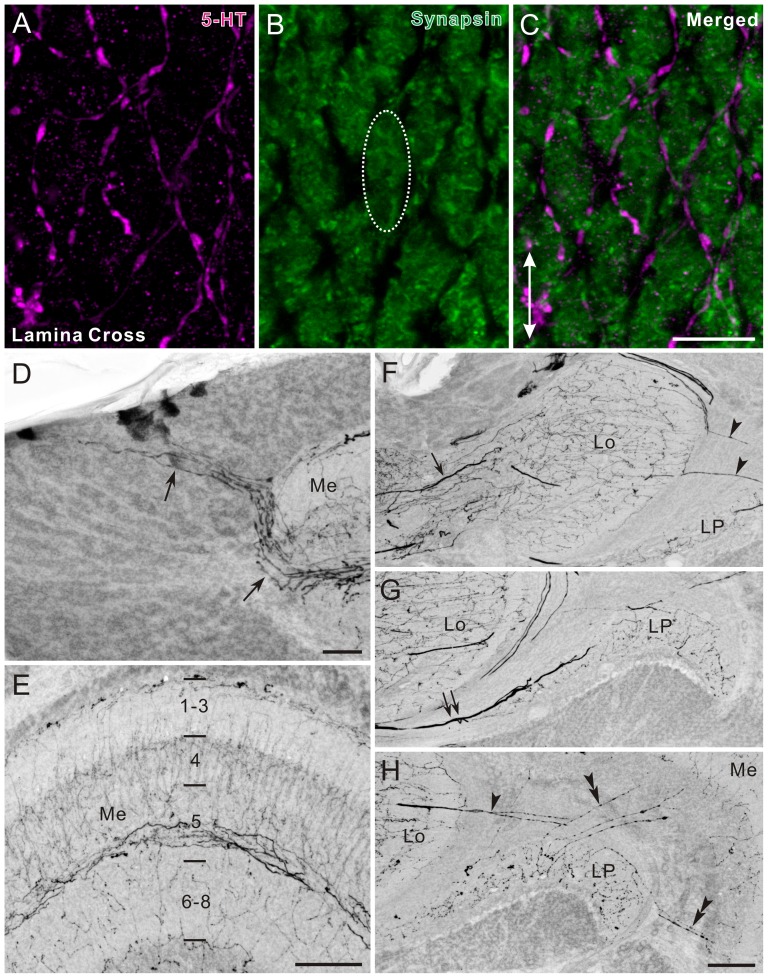
5-HT immunoreactivity in the optic lobe. **A–C**: Cross section through the lamina synaptic layer. 5-HT-positive fibers (magenta in A,C), shown as a reconstruction projected on a single confocal plane of anti-synapsin-labeled cartridges (green in B,C), run between the cartridges. Dotted oval in B indicates a single cartridge profile. Double-headed arrow in C indicates dorso-ventral axis. **D**: Small OL1 cells innervating layer 5 of the medulla (Me, arrows). **E**: Horizontal section through the medulla, supplied with numerous 5-HT-positive fibers. Layers 1–8 exhibit different patterns of labeling. **F–H**: Horizontal sections of the lobula complex (anterior to the top and medial to the left). F: The lobula (Lo) is densely supplied with 5-HT-positive fibers, which originate in the central brain (arrow). Some fibers extend from the lobula to the medulla (arrowheads). G: The lobula plate (LP) is penetrated by 1–2 thick fibers (double arrows). H: Fine arborizations in the lobula plate bear blebby terminals. Double arrowheads indicate fibers linking the lobula plate and medulla. Arrowhead indicates fibers projecting to the proximal medulla. Scale bar = 10 µm in C (applies to A,B); 20 µm in D; 50 µm in E,H (applies to F,G).

The cluster of OL2 neurons appears to contain different types of neurons. However, we could not resolve the complete projection pattern of any single OL2 neuron because of the complexity in their branching pattern. Arising fibers from OL2 cell bodies take at least four distinct pathways (Fig. 4C,D). The first pathway is morphologically centrifugal. Fibers extend from the anterior base of the medulla along the distal surface of the medulla (arrows in Fig. 4A) to the posterior edge of the lamina. They give rise to tangentially oriented processes along the proximal margin of the lamina, which extend arborizations with blebs throughout the lamina neuropil (Fig. 4E). Interestingly the staining pattern differs markedly between the anterior and posterior halves of the lamina. In the posterior part of the lamina, stained processes are concentrated along the periphery of the neuropil (arrowheads in Fig. 4E) whereas the neuropil itself shows fine fibrous staining. In contrast, in the anterior lamina, immunoreactive processes have prominent blebby terminals and extend throughout the neuropil (Fig. 4E). In sections perpendicular to lamina cartridges, a web-like pattern of 5-HT-positive processes surrounds the cartridges, and only few of these fibers invade the cartridges (Fig. 5A–C). The second pathway connects the optic lobe and the central brain. Fibers from OL2 cell bodies run along the distal surface of the lobula and extend toward the central brain (double arrowhead in Fig. 4C), but their terminal sites in the central brain are yet to be identified. Neurons following the third pathway invade layer 5 of the medulla and give rise to numerous tangential processes along the layer (arrow in Fig. 4C). Finally, fibers from some OL2 neurons pass along the anterior margin of the lobula and extend thick branches to the central brain (arrow in Fig. 4D).

The 5HT-immunoreactive OL1 neurons are probably medulla intrinsic or amacrine neurons and project thin fibers into medulla layer 5 (arrows in Fig. 5D).

The medulla is densely supplied with 5-HT-positive fibers (Fig. 5E). At its distal surface, tangentially-oriented blebby fibers were labeled. Layers 1–3 contain thin fibers, diffusely projecting along the medulla columns. Layer 4 is densely innervated by numerous thin fibers, and layer 5 is invaded by thick tangentially oriented processes. Layers 6–8 are supplied with diffusely and widely distributed small fibers that extend from the central brain via the lobula and lobula plate (Fig. 5E–H).

Two pathways connect the optic lobe and the central brain, the anteriorly running optic tracts and the posterior optic tract. 5-HT-positive fibers of the anterior tracts invade the lobula and spread numerous small branches throughout the lobula: no clear stratification is visible there (Fig. 5F). Relatively thick fibers pass through the distal surface of the lobula and extend toward the proximal surface of the medulla (arrowheads in Fig. 5F,H). They ramify and spread numerous small branches in the proximal layers 6–8 of the medulla (Fig. 5E). In another pathway, strongly labeled thick fibers innervate the lobula plate through the posterior optic tract and give rise to many small branches with blebs in all layers (Fig. 5G,H). Some processes pass through the distal surface of the lobula plate and connect the lobula plate and layers 6–8 of the medulla (double arrowheads in Fig. 5H).

### Tyramine

**Figure 6:**
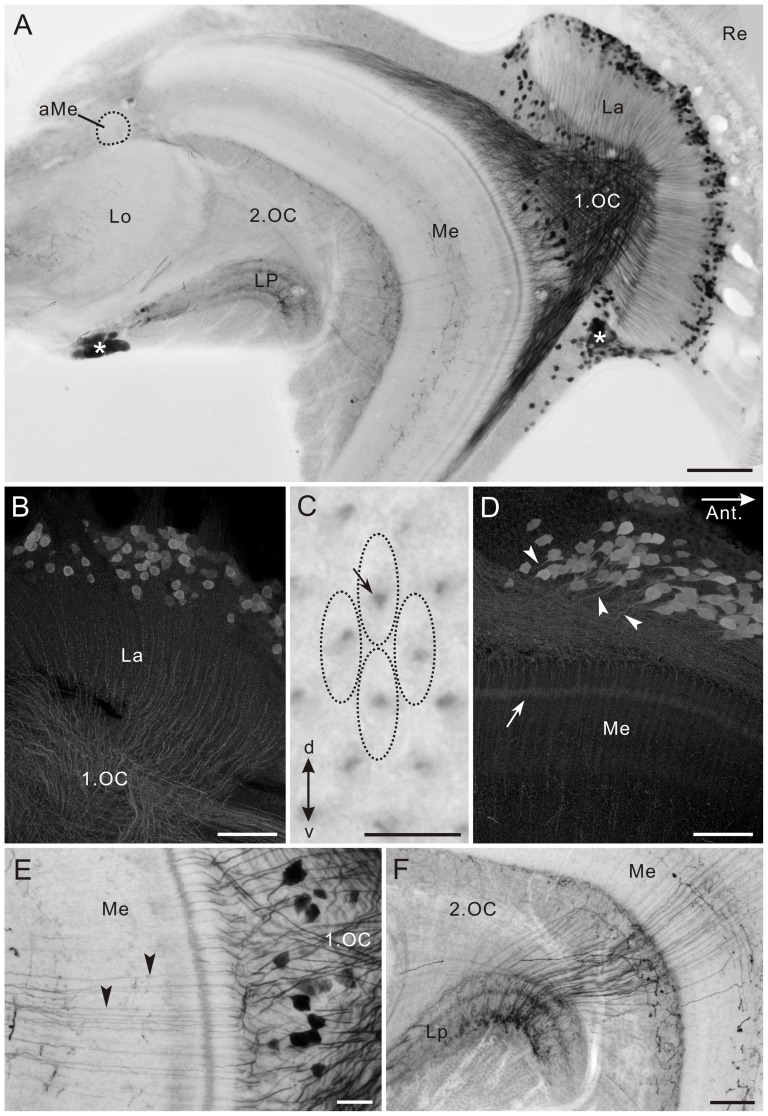
Tyramine immunoreactivity in the optic lobe of *Papilio xuthus*. **A**: Horizontal section. The accessory medulla (aMe) is free of labeling. Asterisks indicate remnants of larval stemmata. **B**: Horizontal section of the lamina (La). Large monopolar cells (LMCs) exhibiting tyramine immunoreactivity project thin axons through the lamina into the medulla. **C**: Lamina cross section with axon profiles of tyramine-positive LMCs (arrow). Each cartridge (dotted ovals) contains one tyramine-positive axon. d, dorsal; v, ventral. **D**: Horizontal section of the anterior medulla. Numerous tyramine-positive neurons probably correspond to medulla amacrine cells. Their primary neurites enter the medulla (arrowheads). Layer 2 exhibits moderate staining (arrow, also see Fig. 7D–F). **E,F**: Horizontal sections. E: Thin fibers from cell bodies in the first optic chiasma (1.OC) pass through the medulla (arrowheads) and terminate in both outer and inner layers of the lobula plate (LP, F). 2.OC, second optic chiasma; Lo, lobula; Re, retina. Scale bar = 100 µm in A; 50 µm in B,D,F; 10 µm in C; 20 µm in E.

Tyramine labeling was detected in the four major visual neuropils but not in the accessory medulla (Fig. 6A). The dark appearance of larval stemmata is due to the pigment cells surrounding the photoreceptors, so any immunoreactivities are uncertain (Fig. 6A). Four distinct types of immunoreactive neurons could be distinguished. Their cell bodies are located 1) in the lamina cell body rind, 2) in the anterior and posterior cell body rind surrounding the first optic chiasma, 3) in the first optic chiasma, and 4) at the anterior median edge of the medulla and lobula.

The first group of neurons is a set of LMCs. Their neurites extend through the lamina synaptic layer and continue through the first optic chiasma to synaptic terminals in the medulla (Fig. 6A,B). LMCs generally have numerous dendritic spines in the lamina [10], [12], [34]–[36], but these could not be detected with anti-tyramine labeling (Fig. 6B). In cross sections of the lamina synaptic layer, a single immunoreactive profile was found in each cartridge (Fig. 6C).

**Figure 7:**
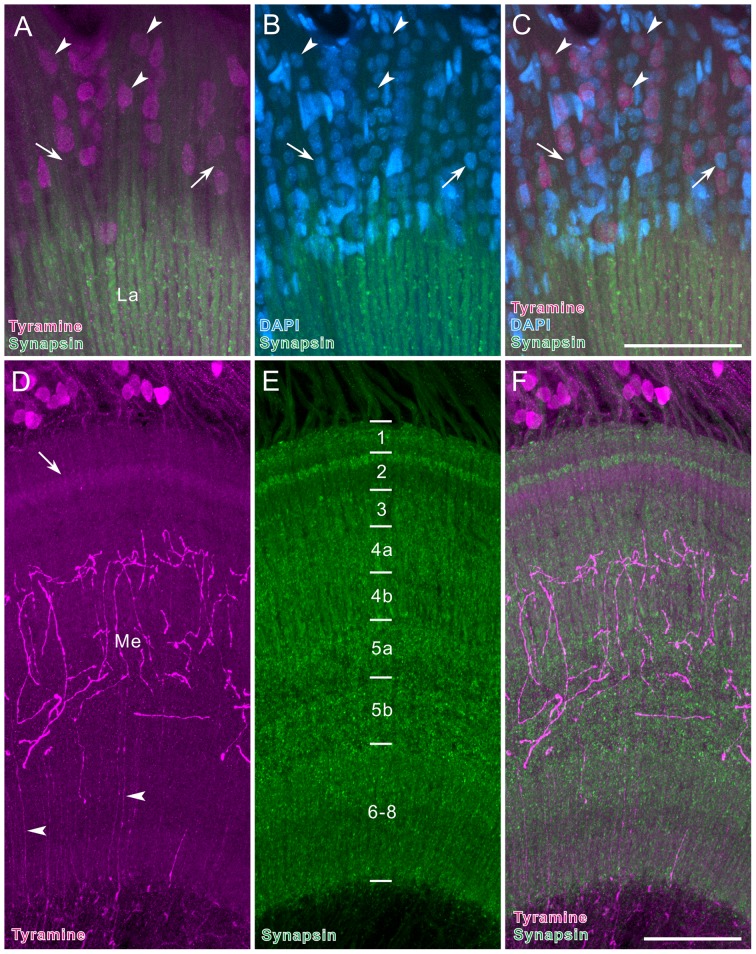
Tyramine immunofluorescence in the lamina and medulla. **A–C**: Single horizontal optical sections through the lamina (La, thickness = 1 µm), stained with anti-tyramine (magenta in A,C), the nuclear marker DAPI (blue in B,C), and anti-synapsin (green in A–C). About one fourth of LMCs were double-labeled with anti-tyramine and DAPI (arrowheads), whereas others were exclusively labeled with DAPI (arrows). **D–F**: Horizontal section of the medulla (Me), double-labeled with anti-tyramine (magenta in D,F) and anti-synapsin (green in E,F). The proximal part of layer 2 exhibits moderate tyramine immunoreactivity (arrow in D), which is attributable to terminals of tyramine-positive LMCs. Layers 4 and 5 are supplied with widely branching tyramine-positive fibers. Thin fibers in layers 6–8 (arrowheads in D) are axons derived from the cell bodies in the first optic chiasma (see also Fig. 6E,F). Numbers in E indicate medulla layers. Scale bar = 50 µm in C (applies to A,B),F (applies to D,E).

Compared to the density of LMCs, revealed in Azur II-stained preparations, that of tyramine-positive LMCs is low (compare Fig. 6B with inset of Fig. 1), implying that not all but a subset of LMCs is tyramine-positive. To evaluate the ratio between tyramine-positive and -negative LMCs, we performed double labeling with anti-tyramine and the nuclear marker DAPI. We found that 44 of 194 LMCs are tyramine-positive on a single optical slice (1 µm thickness), indicating that about one-fourth of the LMCs are tyramine-positive (Fig. 7A–C). To determine where the tyramine-positive LMCs terminate, we examined the medulla double-labeled with anti-tyramine and anti-synapsin antibodies. Double immunofluorescence revealed moderate tyramine immunoreactivity in the proximal part of medulla layer 2 (arrow in Fig. 7D), which most likely corresponds to the terminals of tyramine-positive LMCs.

**Figure 8:**
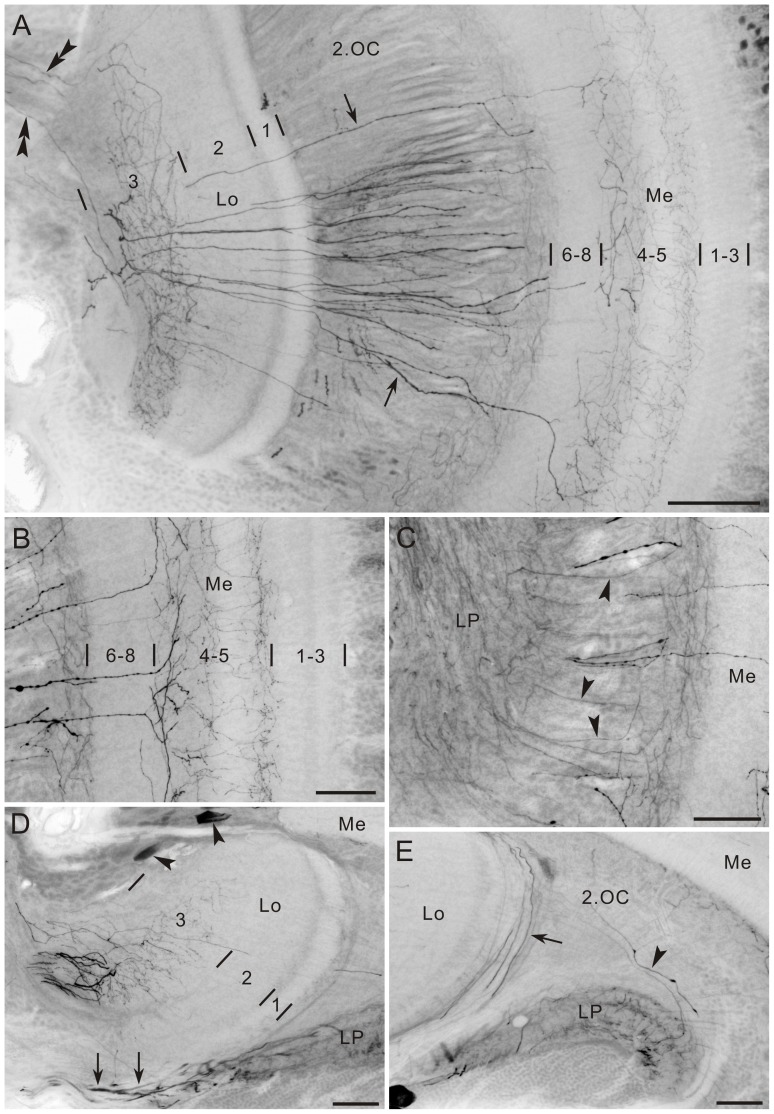
Tyramine immunoreactivity. **A–C**: Frontal sections. A: Putatively centrifugal blebby processes connect the central brain to the lobula (Lo) and medulla (Me). The fibers from the central brain (double arrowheads) invade the lobula and give rise to arborizations in its layer 3. Some of the fibers continue through the second optic chiasma (2.OC, arrows) and innervate medulla layers 4 and 5. B: Processes in medulla layers 4 and 5. C: Connection between the medulla and lobula plate (LP, arrowheads). **D,E**: Horizontal sections of the lobula complex. D: Two tyramine-positive cell bodies are located at the anterior edge of the lobula (arrowheads). The lobula plate is innervated by thick tyramine-positive processes from the central brain (arrows). E: Fibers running along the distal surface of the lobula (arrow) and innervating the lobula plate (arrowhead). Numbers in A, B and D indicate layers. Scale bar = 100 µm in A; 50 µm in B,C,D,E.

The second group of cell bodies, surrounding the first optic chiasma, probably consists of medulla amacrine neurons (Fig. 6A,D) because their primary neurites project toward the distal surface of the medulla (arrowheads in Fig. 6D). The third group of cells in the first optic chiasma probably consists of transmedullary columnar neurons projecting to the lobula plate. They appear to extend thin columnar axons through the medulla without bearing side branches and densely innervate proximal regions of the outer and inner layers of the lobula plate (Fig. 6E,F). The fourth group of cell bodies (arrowheads in Fig. 8D) appears to extend fibers along the distal surface of the lobula (arrow in Fig. 8E).

Layer 3 of the lobula is densely supplied with tyramine-positive processes from the central brain (double arrowheads in Fig. 8A). Sets of thick beaded fibers pass individually through the distal surface of the lobula. They project via the second optic chiasma to the medulla (arrows in Fig. 8A) and ramify widely in layers 4 and 5 (Fig. 8A,B). Fine processes linking the proximal surface of the medulla and the lobula plate were labeled with the anti-tyramine in the second optic chiasma (arrowheads in Fig. 8C). The lobula plate is innervated by processes from the central brain (arrows in Fig. 8D) and by additional fibers of unknown origin (arrowhead in Fig. 8E).

### Gamma-aminobutyric acid (GABA)

All neuropils in the optic lobe including the accessory medulla are supplied with GABA-positive processes, and several types of cell bodies surrounding these neuropils exhibited GABA immunoreactivity. No GABA immunoreactivity was detected in the retina.

**Figure 9:**
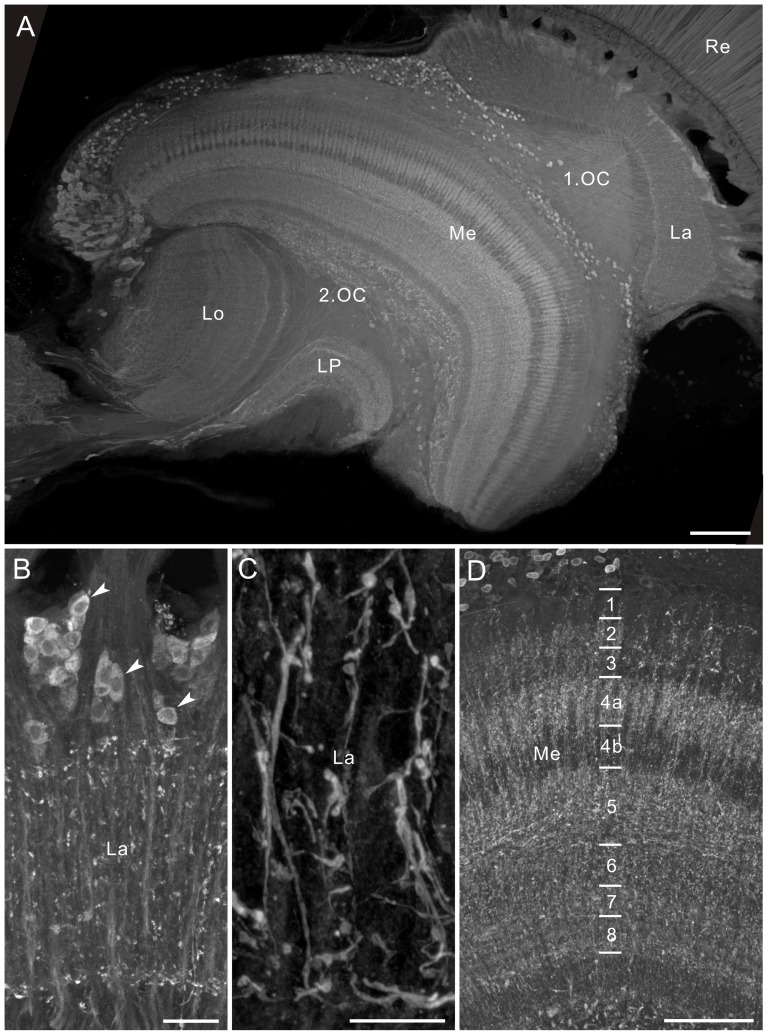
GABA immunofluorescence in the optic lobe of *Papilio xuthus*, horizontal sections. **A**: GABA-positive cell bodies are located in the lamina cell body layer, at the anterior rim of the medulla (Me), along the distal surface of the medulla, and between the medulla and lobula plate (LP). **B**: Longitudinal section of the lamina (La). Some LMCs are GABA-positive (arrowheads). **C**: Enlarged image of the lamina synaptic layer, showing GABA-positive longitudinally-oriented fibers with small collaterals and beaded terminals. **D**: All layers of the medulla are innervated by GABA-positive fibers. 1.OC, first optic chiasma; 2.OC, second optic chiasma; Lo, lobula; Re, retina. Scale bar = 100 µm in A; 20 µm in B; 10 µm in C; 50 µm in D.

**Figure 10:**
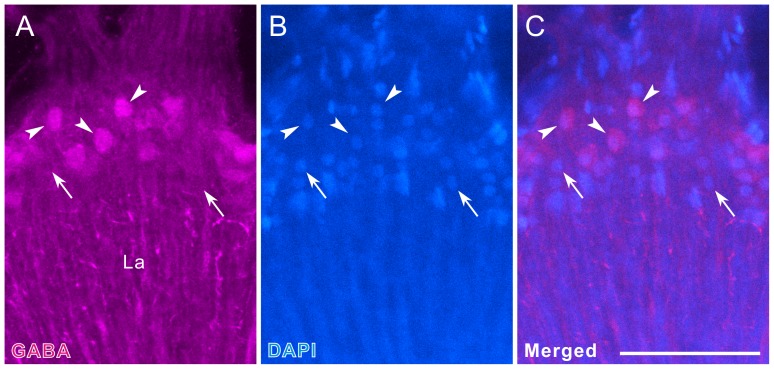
A subset of LMCs immunoreactive to anti-GABA. Horizontal single optical section of the lamina (La, thickness = 1 µm), double-labeled with anti-GABA (magenta in **A,C**) and DAPI (blue in **B,C**). A subset of LMCs is GABA-positive (arrowheads), while others are exclusively labeled with DAPI (arrows). Scale bar = 50 µm in C (applies to A,B).

In the lamina, both cell body and synaptic layers were labeled (Fig. 9A, B). A subset of LMCs displays moderate GABA immunoreactivity (arrowheads in Fig. 9B). The lamina synaptic layer contains many GABA-positive processes, which are uniformly distributed along the longitudinal axes of lamina cartridges (Fig. 9B). These processes often bear short blebby collaterals (Fig. 9C) and might originate from LMCs and centrifugal neurons. GABA-positive LMCs have never been reported in any insects so far. To determine the ratio between GABA-positive and -negative LMCs, we double-labeled tissues with the anti-GABA and DAPI (Fig. 10), which revealed that 36 of 105 LMCs are GABA-positive on a single optical slice (1 µm thickness), indicating that about one-third of LMCs are GABA-positive.

All layers of the medulla show GABA immunostaining (Fig. 9A,D). Layer 1 is supplied by some longitudinally-oriented thin fibers. Layers 2 and 3 exhibit a bushy pattern of labeling. A similar pattern, but denser, is seen in the distal part of layer 4 (4a) in contrast to sparser staining in the proximal part of layer 4 (4b). Layer 5 is predominantly innervated by tangentially oriented fibers, probably originating from large GABA-positive neurons with cell bodies near the anterior rim of the medulla. Layer 6 displays a pattern of fine granular staining. Similar patterns are present in layers 7 and 8, but staining is denser than in layer 6.

**Figure 11:**
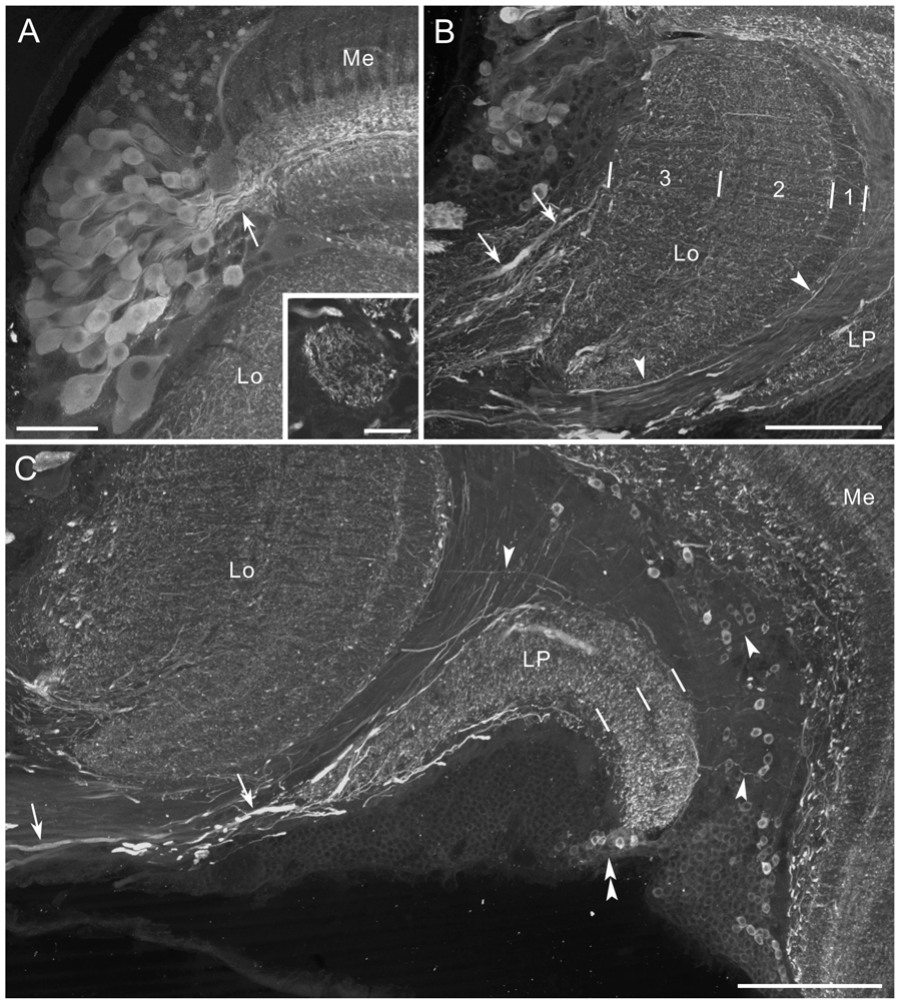
GABA immunofluorescence in the medulla, lobula and lobula plate. Horizontal sections. **A**: A cluster of GABA-positive cell bodies at the anterior rim of the medulla (Me) projects primary neurites into layer 5 (arrow). Inset shows a single optical section containing the accessory medulla, innervated by GABA-positive fibers. **B**: The lobula (Lo) is innervated by GABA-positive thick processes from the central brain (arrows; anterior to the top, medial to the left). The processes bear numerous fine fibers, distributed throughout the lobula. Arrowheads indicate thin fibers running along the distal surface of the lobula. **C**: The lobula plate (LP) is invaded by thick processes (arrows), and both layers are densely innervated by fine fibers. Arrowheads indicate thin fibers linking the medulla, lobula and lobula plate. GABA-positive cell bodies are located between the medulla and lobula plate, and at the posterior margin of the lobula plate (double arrowhead). Scale bar = 50 µm in A; 20 µm in inset of A; 100 µm in B,C.

Numerous GABA-positive cell bodies are distributed around the medulla (Fig. 9A). These neurons are probably either medulla tangential, medulla intrinsic (amacrine), or transmedullary neurons. Large GABA-positive cell bodies at the anterior rim of the medulla extend neurites tangentially into layer 5 of the medulla (arrow in Fig. 11A). Numerous small cell bodies along the distal surface of the medulla are probably columnar elements of the medulla (Fig. 9A). Many small cell bodies, distributed diffusely between the proximal surface of the medulla and the lobula plate, also exhibit GABA immunoreactivity (Fig. 11C).

The lobula is invaded by thick GABA-positive axons from the central brain, which ramify widely in the neuropil (arrows in Fig. 11B): three layers of the lobula were differently labeled. The lobula plate is also innervated from the central brain by GABA-positive fibers with numerous side branches (arrows in Fig. 11C). Both layers of the lobula plate show a fine granular labeling (Fig. 11C). At the posterior edge of the lobula plate, a cluster of small cell bodies was labeled (double arrowhead in Fig. 11C). In the second optic chiasma, fine GABA-positive fibers connect the medulla, lobula, and lobula plate (arrowheads in Fig. 11C).

## Discussion

In the insect optic lobe, a variety of neuroactive substances including amines, amino acids, acetylcholine, and neuropeptides has been mapped by immunocytochemistry [7], [20], [37], [38]. Identification of neuronal cell types that use these substances as neurotransmitters and modulators is necessary for a better understanding of visual information processing in the optic lobe in general. We have analyzed the distribution of three biogenic amines, histamine, serotonin (5-HT) and tyramine, and the amino acid GABA in the optic lobe of *P. xuthus*. All of these substances probably function as neurotransmitters or modulators in the butterfly visual system.

**Figure 12:**
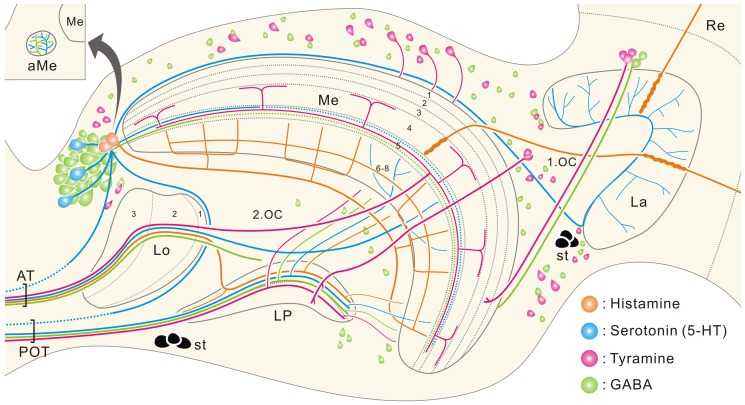
Schematic illustration depicting the distribution of immunolabeled cell bodies and major fiber trajectories. Horizontal plane. Anti-histamine in orange, anti-5-HT in blue, anti-tyramine in magenta, and anti-GABA in green. In the medulla (Me), 5-HT-positive fibers in layers 1–4 and GABA-positive fibers in layers 1–4 and 6–8 are omitted for clarity. In the lobula (Lo) and lobula plate (LP), likewise, immunoreactive arborizations in respective layers are omitted. Solid lines indicate confirmed pathways, and dotted lines are putative pathways. 1.OC, first optic chiasma; 2.OC, second optic chiasma; aMe, accessory medulla; AT, anteriorly running optic tracts; La, lamina; POT, posterior optic tract; Re, retina; st, larval stemmata.

Figure 12 summarizes the present immunocytochemical results. In addition to antibodies against neurotransmitter candidates, we also used an antibody against *D. melanogaster* synapsin to visualize synaptic neuropils. Synapsins belong to a small family of synaptic vesicle-associated phosphoproteins that participate in regulating transmitter release [39], although their exact function is still controversial. The synapsin antibody detects an epitope that is widely conserved in the nervous systems of arthropods including insects [40], crustaceans [41] and spiders [42]. In *P. xuthus*, all visual neuropils show synapsin immunostaining.

Differences in staining intensity allowed us to distinguish eight layers in the medulla, three layers in the lobula, and two layers in the lobula plate; medulla layers 4 and 5 were further divided into two sublayers 4a/4b and 5a/5b, respectively. Based on histological criteria, eight medulla layers are also distinguished in the butterfly, *Pieris brassicae* [36], and ten layers in the monarch butterfly, *Danaus plexippus* [43].

### Photoreceptors

Several lines of histological and pharmacological evidence strongly suggest that histamine is the neurotransmitter of insect photoreceptors. Histamine immunoreactivity has been localized to photoreceptors in several insect species including bees, cockroaches, crickets, flies, and locusts [44]. Hardie [18] demonstrated that iontophoretically applied histamine hyperpolarizes the LMCs, which are postsynaptic to the photoreceptors R1–R6 in the house fly, *Musca domestica*. The histamine receptors appear to be histamine-gated chloride channels [45]. Also in *P. xuthus*, histamine most likely functions as neurotransmitter of photoreceptors because uniform fibrous histamine immunostaining was detected in the lamina synaptic layer, where axons of short visual fibers (R3–R8) terminate, and characteristic terminal specializations in medulla layer 4, corresponding to those of long visual fibers (R1, R2 and R9), were histamine-positive (Fig. 12). We could not identify each profile of the 9 photoreceptor axons per ommatidium in lamina cross sections. However, considering that besides histamine no other neurotransmitters have been identified in insect photoreceptors and that histamine signals are widely distributed within the lamina cartridge profiles (Fig. 2C–E), histamine is the likely transmitter of R1–9 photoreceptors of *P. xuthus*. Histamine immunoreactivity was also found in the remnant larval photoreceptors in the adult optic lobe (asterisks in Figs. 2A, 3A, and 6A).

### Lamina: LMCs

The first visual neuropil, the lamina, is innervated by histamine-, 5-HT-, tyramine-, and GABA-positive processes (Fig. 12).

One of the most important findings in the present study is the occurrence of tyramine- and GABA immunoreactivities in LMCs of *P. xuthus* (Figs. 6A,B, 9B, and 12). So far neither tyramine nor GABA has been proposed to be a neurotransmitter of LMCs in any insects. Tyramine is the precursor of octopamine, a widely occurring neuromodulator in insect nervous systems. Increasing evidence, however, indicates that tyramine itself could also function as a neuroactive substance in insects. In the locust, *Schistocerca gregaria*, tyramine immunoreactivity is detected in octopamine-negative cells [19]. Moreover, in several insects, a range of octopamine receptors is often activated by tyramine as well [21]. At least in *D. melanogaster* a receptor specific for tyramine has been cloned, which does not cross-react with octopamine [46]. These data imply the possibility that tyramine functions as a neurotransmitter in insects including *P. xuthus*. Given that tyramine is the precursor of octopamine, tyramine-positive neurons may fall into two categories; 1) neurons containing both octopamine and tyramine (octopaminergic), and 2) neurons containing tyramine but not octopamine (tyraminergic). Double labeling with anti-tyramine and anti-octopamine is one way to distinguish between these alternatives, but those experiments have not been successful in *P. xuthus* yet. At any rate, tyramine or octopamine is a novel transmitter candidate of LMCs, following acetylcholine and glutamate in flies [47]–[50], glutamate in honeybees [48], [51] and cockroaches [48], and a peptide related to Mas-allatotropin [52] and nitric oxide [53] in locusts.

We estimated that each cartridge contains a single tyramine-positive LMC axon, and that about a quarter of all LMCs are tyramine-positive (Figs. 6C, and 7A–C). The morphologies of LMCs have been described in the Australian Orchard butterfly, *Papilio aegeus*, by Golgi impregnation [34]: there are four morphologically different types of LMCs, termed L1–L4. They differ in both branching patterns in the lamina and terminal layers in the medulla. Among the four types, L3 extends a small number of short dendritic spines within the cartridge and terminates in medulla layer 3, corresponding to the proximal part of medulla layer 2 in *P. xuthus* where tyramine-positive LMCs terminate (Fig. 7D). The tyramine-positive LMCs also appear to bear a few spines in the lamina (Fig. 6B). According to these morphological properties, the tyramine-positive LMCs of *P. xuthus* may be the counterpart of L3 of *P. aegeus*.

GABA, a major neurotransmitter at inhibitory synapses [54], appears to be another transmitter candidate of LMCs. Two types of GABA receptors are known in insects: Ionotropic GABA_A_ receptors directly coupled to chloride channels [55] and metabotropic GABA_B_ receptors coupled to G-proteins [56]. In *D. melanogaster*, cells expressing transcripts for the GABA synthesizing enzyme glutamic acid decarboxylase 1 as well as subunits of both GABA_A_ and GABA_B_ receptors are distributed throughout the brain including the optic lobe [57], suggesting the physiological importance of GABA in neural circuits. Olfactory receptor neurons (ORNs) synapse upon projection neurons in each glomerulus of the antennal lobe and GABA is physiologically demonstrated to be involved in inhibitory interactions needed for odor-evoked synchronization of projection neuron activity and for interglomerular inhibition [58]. In *D. melanogaster*, ORNs are suggested to express both GABA_A_ and GABA_B_ receptors, which play an important role in presynaptic inhibition [59]. GABA_B_ receptor expression levels in ORNs are also a determinant of glomerulus-specific olfactory gain control [60]. Circadian pacemaker neurons of *D. melanogaster* also require GABA_B_ (R3 subtype) receptor-mediated inhibitory input to generate 24-hour locomotor rhythms [61]. To date, however, no physiological data are available on the role of GABA in the optic lobe except for pharmacological and anatomical evidence of GABAergic inhibitory inputs to fly tangential cells in the lobula plate [62], [63]. Inhibitory interactions are essential to explain certain visual phenomena, such as lateral inhibition and color opponency. GABA-positive LMCs might be involved in such inhibitory interactions. Double labeling with DAPI and anti-GABA indicated that one-third of LMCs are GABA-positive (Fig. 10). If each cartridge of *P. xuthus* contains four LMCs as in *P. aegeus*, some cartridges could be innervated by more than two GABA-positive LMCs. Because neither branching patterns nor terminal regions were detected, we could not assign the GABA-positive LMCs to any type of LMCs of *P. aegeus* at the present stage.

### Lamina: centrifugal neurons

The lamina appears to be under control of centrifugal neurons containing 5-HT and GABA. In many insects studied so far, the lamina is innervated by 5-HT-positive processes, derived from cell bodies either along the anterior edge of the medulla or in the central brain [33], [64]. In flies, the lamina is innervated by a pair of neurons that have cell bodies in the central brain, connecting the lamina to the medulla, lobula and lobula plate [64]. In *P. xuthus*, as in the sphinx moth *M. sexta* [33], the lamina is innervated by a population of neurons with large cell bodies located at the anterior rim of the medulla (Fig. 12). Not only the origin of 5-HT-positive fibers in the lamina, but also the pattern, by which the lamina is innervated, is diverse among species. In ants, flies, mantises, and honeybees, arborizations are restricted to certain sublayers [64]–[66]. In contrast, in the sphinx moth *M. sexta*, 5-HT-positive processes are homogeneously distributed throughout the lamina [33]. The distribution pattern is unique in *P. xuthus*; the anterior half of the lamina is innervated more prominently than the posterior half (Fig. 4E). Whether and how this pattern is crucial for visual signal processing remain to be explored.

5-HT-positive varicose terminals in the lamina were investigated by immuno electron microscopy in the blowfly, *Calliphora erythrocephala* [65]. That study shows that 5-HT immunoreactivity is concentrated in large granular and small clear vesicles. In *P. xuthus*, 5-HT-positive processes are predominantly distributed around the cartridges, but hardly within them (Fig. 5A–C). In this region 5-HT might be mainly released between cartridges in non-synaptic manner. 5-HT is known to be involved in circadian clock systems [67], [68]. The LMCs of the house fly *M. domestica* change their axon diameters in a circadian cycle, which is modified by 5-HT injection [67]. 5-HT also affects the circadian rhythm in the electroretinogram in the blowfly *C. erythrocephala* [68]. Taken together, 5-HT appears to be involved in diurnal regulation of visual interneurons in insects, which has also been implied electrophysiologically in *P. xuthus* [69]. 5-HT also modulates *Shaker* potassium channels in *D. melanogaster* photoreceptors [70].

GABA-positive neurons provide centrifugal inputs to the lamina [7], [47]. In *D. melanogaster*, transmedullary C2 and C3 neurons, which have cell bodies proximal to the medulla, collaterals in the medulla and terminals in the lamina, are immunoreactive to anti-GABA and anti-glutamic acid decarboxylase antibodies [47]. Corresponding GABA-positive neurons have been also reported in other flies [71]–[73], cockroaches [74], and moths [32]. The GABA-positive cell bodies, which are diffusely distributed proximal to the medulla in *P. xuthus* (Figs. 11C, and 12), are probably the counterparts of the C2 and C3 neurons. Another set of GABA-positive centrifugal inputs is found in a cockroach [74] and in a locust [75]. Those neurons have cell bodies at the anterior rim of the medulla and bear tangential processes in the proximal lamina. GABA-positive cell bodies at the anterior rim of the medulla in *P. xuthus* (Figs. 11A, and 12) may, likewise, project axonal fibers to the lamina.

### Medulla

The medulla is the largest neuropil in insect cephalic ganglia, containing a large variety of transmitter candidates [7], [20], [37]. The medulla of *P. xuthus* is innervated by numerous immunoreactive processes (Fig. 9D), which appear to be derived from columnar, amacrine, and tangential neurons as in many other insects studied [7], [20], [37]. GABA-positive columnar neurons with cell bodies along the distal surface of the anterior medulla are present in a cockroach [74] and in a moth [7], [32]. Also in flies, GABA-positive medulla columnar neurons link the medulla to the lobula and lobula plate [71]. Likewise, the large number of small GABA-positive cells along the distal surface of the medulla in *P. xuthus* (Figs. 9A, and 12) possibly provides columnar axons in the medulla.

In the locust, *S. gregaria*, a population of medulla amacrine cells, whose cell bodies are located around the first optic chiasma, shows octopamine immunoreactivity [7], [19]. Therefore, tyramine-positive cell bodies in the corresponding region of *P. xuthus* (Figs. 6A,D, and 12), are most likely those of medulla amacrine cells, and could contain octopamine. A small number of transmedullary neurons with cell bodies in the first optic chiasma and terminals in the lobula plate, exhibits tyramine immunoreactivity in *P. xuthus* (Figs. 6E,F, and 12). They appear to have no collaterals in the medulla. A similar type of transmedullary neurons, Tm23 and Tm24, bearing arborizations only in the lobula, have been identified in *D. melanogaster* [12]. These two types of neurons may function as amacrine or local interneurons. Other putative medulla amacrine cells of *P. xuthus* are the OL1 and a subset of OL2 neurons (Fig. 4B). Corresponding 5-HT-positive neurons in the sphinx moth *M. sexta* (OL1) and in flies (similar to OL2) have likewise been proposed to be medulla amacrine neurons [20], [33]. Histamine-positive neurons with cell bodies at the anterior rim of the medulla and arborizations in the distal medulla, may also be amacrine cells (Figs. 3A,B, and 12).

GABA-positive cell bodies at the anterior rim of the medulla in *P. xuthus* (Figs. 11A, and 12) may be those of tangential neurons, extending fibers to the central brain in addition to the tangential processes in the medulla. In fact, sets of medulla tangential neurons with fibers projecting to the central brain show GABA immunoreactivity in flies and honeybees [71]. Alternatively, some of these neurons may be medulla amacrines.

Like the lobula and lobula plate, the medulla of *P. xuthus* is innervated by centrifugal neurons with cell bodies in the central brain (Figs. 8A, and 12). Layers 4 and 5 are innervated by tyramine-positive centrifugal processes, which bear numerous branches in the lobula before entering the medulla (Fig. 8A). Similarly the proximal layers of the medulla are innervated by 5-HT-positive fibers, which arborize in the lobula and lobula plate before entering the medulla (Fig. 12). A prominent set of such centrifugal neurons is the octopamine-positive protocerebral-medulla 4 (PM4) neurons in the locust, *Locusta migratoria* [76], [77]. Two of these neurons have cell bodies near the oesophageal foramen and dendritic arborizations in the antennal mechanosensory and motor center and in various regions in the median protocerebral neuropil, and send extensive axonal projections into the lobula and medulla [76], [77]. PM4 neurons respond to a wide variety of sensory inputs including tactile, acoustic and visual stimuli, which all dishabituate DCMD neurons that are involved in visually-guided collision avoidance responses. The dishabituation of DCMD neurons can, therefore, be regarded as an arousal mechanism of the animal, mediated by octopamine released from PM4 neurons [77].

Similar neurons also exist in cockroaches and honeybees [78], and in flies [79], suggesting similar arousal mechanisms in these species as well. Tyramine-positive centrifugal processes connecting the central brain to the lobula and medulla in *P. xuthus* (Figs. 8A, and 12) might be derived from counterparts of the locust PM4 neurons.

### Accessory medulla

The accessory medulla is a small neuropil at the anterior edge of the medulla. It is the remnant of the larval visual center [80] and the site of the master circadian pacemaker in cockroaches and flies [81], [82]. The accessory medulla of *P. xuthus* is immunoreactive with 5-HT and GABA antisera (Figs. 4B, and inset of 11A), but neither with the histamine- nor with the tyramine antiserum (Figs. 3B, and 6A). In contrast, the accessory medulla of the cockroach *Periplaneta americana* is innervated by histamine-positive fibers, derived from cell bodies close to it and also from photoreceptors at the posterior portion of the compound eye [44]. The GABA-positive fibers in the accessory medulla of *P. xuthus* are described previously [80]. Both 5-HT- and GABA-positive fibers in the accessory medulla are probably derived from sets of immunoreactive cell bodies at the anterior rim of the medulla.

### Lobula and lobula plate

The lobula and lobula plate are innervated by centrifugal immunoreactive neurons in the central brain (Fig. 12). In the lobula, 5-HT- and GABA-positive processes innervate all layers (Figs. 5F, and 11B), while histamine- and tyramine-positive fibers innervate predominantly layers 2–3 (Fig. 3B) and layer 3 (Fig. 8A,D), respectively. These neurons probably provide inhibitory feedback or modulate signal processing in the neuropil.

The lobula plate, which serves a role in directionally sensitive motion-detection in flies [4]–[6], is innervated by centrifugal processes from the central brain (Fig. 12). Both layers of the neuropil show histamine- (Fig. 3B), 5-HT- (Fig. 5G,H), tyramine- (Fig. 8D,E) and GABA immunoreactivities (Fig. 11C), except for the inner layer that lacks histamine immunoreactivity. In the house fly *M. domestica*, two centrifugal neurons sensitive to horizontal motion (CH-neurons) and a pair of bilateral cells sensitive to vertical motion (V2 or V3) in the lobula plate exhibit GABA immunostaining [71]. In *P. xuthus*, motion-sensitive neurons have likewise been demonstrated in the lobula plate [83], but their neurotransmitter has not been characterized.

### Conclusions and perspectives

The distribution of immunostained neurons summarized in Figure 12 is largely similar to that found in other species [7], [20], [37]. Although the conclusive demonstration of these substances as neurotransmitters or modulators in *P. xuthus* requires further physiological analyses, the present data serve as an important basis to understand the neural mechanisms underlying visual information processing. Special mention is necessary for the following two points. First, the photoreceptors are probably histaminergic as in other insects. Second, lamina LMCs appear to be heterogeneous in their transmitter phenotypes: tyramine (or octopamine) and GABA are now strong transmitter candidates of the LMCs in *P. xuthus*. Given that GABA generally acts at inhibitory synapses, the putative GABAergic LMCs could be a key component underlying any kind of opponency.

We have previously shown that *P. xuthus* can discriminate colors of a target of about 1° visual angle [27]. This is equivalent to the spatial resolution limit predicted from the eye optics. This ability suggests that single or a few retinotopic modules viewing the target bear mechanisms for wavelength processing that require spectral opponency. We have also shown that the nine photoreceptors (R1–R9) embedded in an ommatidium are spectrally heterogeneous [29] and together project into a corresponding single cartridge, where they make mutual contacts and synapse upon LMC spines [84]. A set of photoreceptors with different spectral sensitivities, and both excitatory and inhibitory LMCs are in principle sufficient to produce spectral opponency at the lamina and/or medulla level. Whether and how our findings are linked to any specific properties of the color vision network in *P. xuthus* remains to be addressed.
